# Going Beyond the Millennium Ecosystem Assessment: An Index System of Human Dependence on Ecosystem Services

**DOI:** 10.1371/journal.pone.0064581

**Published:** 2013-05-22

**Authors:** Wu Yang, Thomas Dietz, Wei Liu, Junyan Luo, Jianguo Liu

**Affiliations:** 1 Center for Systems Integration and Sustainability, Department of Fisheries and Wildlife, Michigan State University, East Lansing, Michigan, United States of America; 2 Environmental Science and Policy Program, Department of Sociology and Animal Studies Program, Michigan State University, East Lansing, Michigan, United States of America; MESC; University of South Alabama, United States of America

## Abstract

The Millennium Ecosystem Assessment (MA) estimated that two thirds of ecosystem services on the earth have degraded or are in decline due to the unprecedented scale of human activities during recent decades. These changes will have tremendous consequences for human well-being, and offer both risks and opportunities for a wide range of stakeholders. Yet these risks and opportunities have not been well managed due in part to the lack of quantitative understanding of human dependence on ecosystem services. Here, we propose an index of dependence on ecosystem services (IDES) system to quantify human dependence on ecosystem services. We demonstrate the construction of the IDES system using household survey data. We show that the overall index and sub-indices can reflect the general pattern of households' dependences on ecosystem services, and their variations across time, space, and different forms of capital (i.e., natural, human, financial, manufactured, and social capitals). We support the proposition that the poor are more dependent on ecosystem services and further generalize this proposition by arguing that those disadvantaged groups who possess low levels of any form of capital except for natural capital are more dependent on ecosystem services than those with greater control of capital. The higher value of the overall IDES or sub-index represents the higher dependence on the corresponding ecosystem services, and thus the higher vulnerability to the degradation or decline of corresponding ecosystem services. The IDES system improves our understanding of human dependence on ecosystem services. It also provides insights into strategies for alleviating poverty, for targeting priority groups of conservation programs, and for managing risks and opportunities due to changes of ecosystem services at multiple scales.

## Introduction

The Millennium Ecosystem Assessment (MA) was designed to assess the consequences of ecosystem change and provide scientific information that could aid in sustainably managing ecosystems for human well-being [1]. Although the intended audience was decision-makers, MA also provided a conceptual framework for studying interactions among four key components (i.e., indirect drivers, direct drivers, ecosystem services, and human well-being) of coupled human and natural systems (CHANS) [2], and identified future research needs [3].

Of the interactions between the four components in the MA framework, the linkage between ecosystem services and human well-being is perhaps least understood. The relationship between human well-being and the social factors that influence it has been extensively studied [4], [5], [6], [7], [8]. Through efforts like land change science and structural human ecology, the relationships between changes of ecosystems services and factors that influence them have also begun to be documented from local to regional and global scale [9], [10]. It has been recognized that humans substantially depend on ecosystem services, which range from basic provisions of food, fresh water and fuel, through regulation of water and air quality, to cultural services like ecotourism [1], [11]. The MA also established that during recent decades, two thirds of these services have degraded or are in decline due to the unprecedented scale of human activities [1]. But what are the consequences of such dramatic degradation to short-term and long-term human well-being? The risks of ecosystem degradation and their consequences for human well-being, including nonlinear or abrupt changes, are poorly quantified. On the one hand, there is a lack of a robust theory that links ecological diversity to ecosystem dynamics and ecosystem services [3]. On the other hand, the scientific community lacks understanding of how and to what extent humans depend on ecosystem services. For instance, it has been widely recognized that the poor are most dependent on ecosystem services and most vulnerable to the degradation of ecosystem services [1]; however, it is generally not known how such dependence differs across time, space and various population groups (e.g., across income levels). To better understand, monitor and manage such dependences, a quantitative approach is urgently needed.

From our perspective, there are at least four reasons to quantify human dependence on ecosystem services. First, the relationship between ecosystem services and poverty seems obvious but the dependence of the poor on ecosystem services is rarely quantified, which leads to a pervasive tendency to overlook it in statistics, poverty assessments and natural resource management decisions [12]. A quantitative measurement of such dependence and its integration into decision making could reverse inappropriate strategies that could otherwise lead to further marginalization of the poor and increased pressure on ecosystem services.

Second, benefits provided by ecosystem services are often unequally distributed across different population groups and there may be trade-offs among groups. With better understanding of the distribution of benefits from ecosystem services (e.g., fuelwood, clean water, non-timber forest products, and tourism) across different population groups, conservation and development programs may be better designed to guide the flow of benefits from ecosystem services to target priority population groups. The Wolong Nature Reserve of China provides a compelling example. Most benefits obtained from ecotourism flow to the outside tourism development companies rather than local households [13], [14], a common phenomenon in many other areas [15]. Government policies should encourage household relocation closer to tourism facilities and provide more support to local households (e.g., provide training to improve human capital and offer favorable loan opportunities) to enhance their capacity for participating in tourism businesses [13], [14]. In doing so, more benefits from tourism would flow to local households and substantially reduce their pressure on provisioning services by which local ecosystems provide fuelwood, bamboo shoots, and traditional Chinese medicine.

Third, a better understanding of such dependence would draw attention to currently unmanaged risks and unrealized opportunities that come with ecosystem change. For example, agricultural supply chains can be tightly dependent on ecosystem services and thus are vulnerable to dramatic ecosystem degradation. Unprecedented human activities would likely lead to more frequent extreme climate events and natural disasters (e.g., storms, floods, droughts, and landslides), cause tremendous destruction to ecosystems and their services, and threaten the livelihoods of those people who are highly dependent on corresponding ecosystem services [1], [10]. Yet few managers or policy analysts understand this dependence and related unintended consequences, and even fewer manage the potential risks and opportunities [16], [17].

Fourthly, a quantitative measurement of such dependence would improve the understanding of human-nature interactions. One of the major advances and challenges of the CHANS approach for studying human-nature interactions is to construct coupled models by integrating sub-models of both human and natural subsystems [18]. The key of such integration requires good understanding of the interactions between human and natural subsystems. Currently, there are few coupled models integrating drivers, ecosystem services, and human well-being to systematically understand human-nature interactions [3], [19], [20]. The quantification of human dependence on ecosystem services could potentially serve as a proxy to facilitate such integration and understanding.

The objectives of this study were to (1) propose the conceptual basis of an index of dependence on ecosystem services (IDES) system to measure the degree of human dependence on ecosystem services; (2) demonstrate the construction of the IDES system with empirical data; and (3) illustrate advantages and applications of the proposed IDES system. Specifically, we first provided the conceptual basis of an IDES system, including an overall index and sub-indices for different categories of ecosystem services based on the widely accepted MA framework. We then delineated the process of estimating the indices at Wolong Nature Reserve. We examined temporal changes of the overall IDES and shifts in of the structure of the IDES system (i.e., changes of sub-indices). We compared the overall index with an alternative indicator (i.e., the commonly used agricultural income share) to illustrate the advantages of our proposed index system. Moreover, we assessed the dependence of the poor on ecosystem services. In particular, we analyzed how households' dependences on ecosystem services differ across different degrees of access to capitals (i.e., natural, human, financial, manufactured, and social capitals). We also evaluated the spatial heterogeneity of the overall IDES.

## Methods for Developing an Index System of Human Dependence on Ecosystem Services

### 2.1 Conceptualization of the index system

The term ecosystem services is defined and used in a variety of ways [1], [11], [21], [22], [23], [24], [25]. Here we aligned with the definitions of the MA as the benefits that people obtain directly or indirectly from ecosystems, including both natural systems or highly managed systems [1]. In particular, we included agricultural products as part of ecosystem services. We acknowledge that some literatures might exclude products from highly managed systems (e.g., agro-ecosystems and constructed wetlands) and restrict ecosystem services to goods and services provided by natural systems only. But since the logic of our analysis is driven by the MA, we felt it appropriate to adhere to the definition by the MA. Our proposed index system of dependence on ecosystem services includes an overall index and three sub-indices. The overall index of human dependence on ecosystem services is defined as the ratio of net benefits obtained from ecosystems to the absolute value of total net benefits that derived from ecosystems and other socioeconomic activities (e.g., migrant work, and small business unrelated to ecosystem services, see Table S1). In addition to the overall index, a sub-index can be calculated for each category of ecosystem services under the MA framework (i.e., provisioning, regulating services, and cultural services) [1]. Because supporting services are the bases for other three types of services, following the common practice in ecosystem service assessment, they are not included in IDES to avoid double accounting. As shown by the definition, the higher value of the overall index or sub-index represents the higher dependence on the corresponding ecosystem services, and thus the higher vulnerability to the degradation or decline of corresponding ecosystem services. The equations for calculating three sub-indices and the overall IDES are given as below.
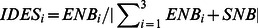
(1)

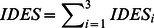
(2)where *i* is the category of ecosystem services (i.e., provisioning, regulating, and cultural services); IDES*_i_* is the sub-index for category *i*; ENB*_i_* is the total net benefit obtained from category *i* ecosystem services; SNB is the total net benefit obtained from socioeconomic activities; IDES is the overall index.

There are four reasons for using net benefits instead of gross benefits. First, ecosystems generate both services and dis-services to humans. Dis-services may include pests and diseases causing reduction in agricultural production and other unintended negative health consequences for organisms including humans [26]. Second, the generation and delivery of ecosystem services may entail costs (e.g., costs of seeds, fertilizers, and pesticides for agricultural products). Using the gross benefits could potentially mislead decision making [27]. One might opt for a program that has the largest increase in gross benefits when another program has a larger yield of net benefits, thereby choosing an inefficient program. Third, using net benefits allows the inclusion of trade-offs between different ecosystem services [28]. Such trade-offs would not be correctly represented if gross benefits are used without considering the costs of delivering those services. Finally, using net benefits facilitates cross-context comparisons. Few previous ecosystem service assessments have evaluated net benefits [29], [30], [31]. Many previous studies have evaluated only the gross benefits so results from different studies are not comparable because ecosystem dis-services and costs of generating ecosystem services can be substantial and vary considerably across contexts.

Both the sub-indices and overall index can be negative. This is because net benefits are not necessarily positive. Total net benefits from each category and all categories of ecosystem services summed can be negative. The ecological and economic meaning of an index with negative value is that the gross benefit obtained from ecosystem services is lower than the sum of costs for generating the corresponding ecosystem services and costs of ecosystem dis-services. For example, the gross benefits of producing agricultural products may be lower than the total costs of seeds, fertilizers, and pesticides.

### 2.2 Methods for constructing the index system

The index system is constructed to assess net benefits of a unit of analysis (e.g., household). The procedures for this approach are in some ways similar to that of many Cost-Benefit Analyses (CBA) [32], [33] and Ecosystem Service Assessments (ESA) [1], [28], [30], [31] where data from a variety of sources are aggregated into an integrated assessment and where the unit of analysis for which the calculation is done must be specified. For CBA and ESA this is often a region or nation, while here we will work at the household level.

Where markets for the gross benefits and costs exist, assessments are relatively straightforward and simple. It is easy to apply market-based valuation methods such as the market price method, the appraisal method, and the avoided cost method [31], [34], [35], [36]. Otherwise, when market data are not available, nonmarket valuation methods such as the contingent valuation method, the travel cost method, the stated preference method, and the hedonic price method can be used [31], [34], [36], [37].There are also cross-cutting methods, such as the benefit transfer method and unit-day value method, which combine both market-based and nonmarket methods [38], [39], [40]. Recently, integrated approaches such as the Integrated Valuation for Ecosystem Services and Tradeoffs (InVEST) have focused on assessing ecological production and then applying economic valuation methods [28], [41]. A variety of reviews and guidelines have discussed these economic valuation methods in detail (e.g., [32], [33], [34], [42]). A summary and critique of the use of these methods was presented by Bateman [37] and thus we do not discuss the use of these economic valuation methods in detail here. We provided an example of how different types of data could be collected through various economic valuation methods to assess the net benefits for constructing the IDES system. The following empirical study will demonstrate the integration of different data sources and valuation methods in detail.

Consider a rural household living in a forest area as an example. Costs and benefits from agricultural products and other socioeconomic activities are parts of the household's income and expenditures and could be captured in a survey with relative ease, using best practices for economic surveys. But when benefits or avoided costs that do not involve market transaction (e.g., non-timber forest products such as fruits, herbal medicine, and fuelwood), they are not shown in the household's income and expenditures as conventionally defined and thus are not captured by conventional economic survey methods. If there are established payments for ecosystem services (PES) programs, then the obtained benefits (e.g., payments) and associated costs (e.g., labor costs for monitoring forests) have market values. If such PES programs are not in place, an ESA can be conducted by adding corresponding survey questions, for example by using contingent valuation method (see case studies in [31], [43]). An ESA can also be conducted using integrated tools such as InVEST for the entire study area (see case studies in [28], [41]) and then disaggregating to the household level (e.g., divided by total number of households in the entire study area or calculated by defining a buffer zone of accessibility to certain ecosystem services based on each household's location, see an example of fuelwood collection in [44]).

## Empirical Demonstration of Constructing the Index System

### 3.1 Description of the demonstration area

**Figure 1:**
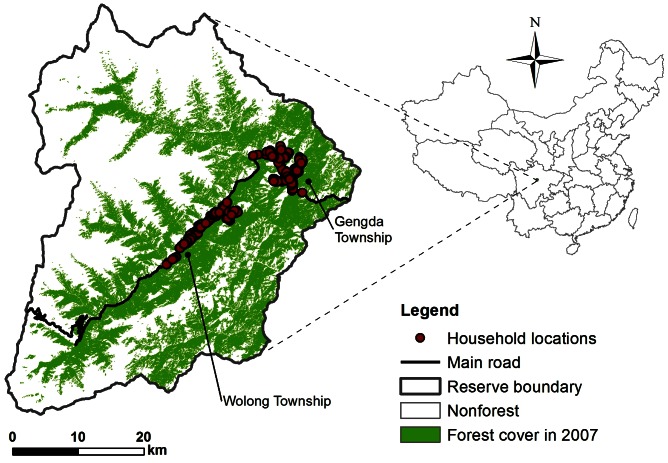
Wolong Nature Reserve in Sichuan Province, southwestern China.

Here we provide an example to demonstrate the index system at Wolong Nature Reserve (N 30°45′–31°25′, E 102°52′–103°24′, Fig. 1) in China. We choose Wolong Nature Reserve as our study area for three reasons. First, situated in the transition from Sichuan Basin to the Qinghai-Tibet Plateau, it is within one of the top 25 global biodiversity hotspots endowed with enormous ecosystem services [45], [46]. Second, it is one of the earliest nature reserves established in China [47]. Like many other protected areas, there are human residents living inside who depend on many types of ecosystem services. Third, our research team has been conducting studies in this area over the past 18 years and has accumulated extensive datasets and local knowledge that give us a well-grounded basis for testing the IDES concept, methods and applications.

The primary purpose of Wolong Nature Reserve is to protect giant pandas (*Ailuropoda melanoleuca*) as well as regional forest ecosystems and rare plant and animal species [48]. When it was established in 1963, its initial size was ∼20,000 ha but was expanded to its current size of ∼200,000 ha in 1975 [48]. It is home to ∼10% of the total wild giant panda population [48]. Currently, there are ∼4900 local human residents, distributed in ∼1200 households in two townships (i.e., Wolong and Gengda Townships) within the Reserve (Fig. 1). The majority of local residents are farmers involved in subsistence activities such as cultivating maize and vegetables, raising livestock (e.g., pigs, cattle, yaks, and horses), collecting traditional Chinese medicine, keeping bees, and collecting fuelwood for cooking and heating (Table S1).

In response to the massive droughts in 1997 and floods in 1998, the Chinese government started to implement a series of ecosystem service policies [49], [50], including two of the world's largest payments for ecosystem services (PES) programs: the Natural Forest Conservation Program (NFCP) and the Grain-to-Green Program (GTGP) [49]. These PES programs aim mainly to improve regulating services such as soil erosion control, water conservation, carbon sequestration, and air purification. From 2000, Wolong Nature Reserve started to implement GTGP, NFCP, as well as a local PES program called the Grain-to-Bamboo Program (GTBP) [48], [51]. NFCP aims to conserve and restore natural forests through logging bans, afforestation, and monitoring, using PES approach to motivate conservation behavior [49], [51]. GTGP and GTBP aim to convert cropland on steep slopes to forest/grassland, and bamboo forest by providing farmers with subsidies, respectively [48], [49], [51].

### 3.2 Data and methods

#### Ethics Statement

The household survey data used here were collected with the permission from the Wolong Administration Bureau of Wolong Nature Reserve. A verbal consent process from interviewees was used due to the low level of education of our interviewees. The verbal consent script was read to the selected interviewees before conducting the survey. Only when they agreed to participate in our survey, we then continued to ask questions in the designed survey instruments. Or else, we did not collect any information but switched to the next selected interviewee. The Institutional Review Board of Michigan State University (http://www.humanresearch.msu.edu/) approved the verbal consent process, verbal consent script, and survey instruments.

#### Data collection and analyses

For this study we used household survey data to estimate the obtained net benefits from ecosystem services (or equivalently gross benefits and costs) for households. This allows us to construct the IDES estimates for households. Our surveys were conducted in the summer of 1999 and the end of 2007 to obtain data covering activities in 1998 and 2007. We tracked the same randomly sampled 180 households so the data constitutes a panel. Usually the household heads or their spouses were chosen as interviewees because our past experience indicates that they are the decision makers and are most familiar with household affairs [52]. To facilitate cross-context comparisons, we used the categories for household income and expenditure data that are consistent with those of the National Bureau of Statistics of China [53] and thus with standard economic survey methods. We used the MA classification for ecosystem services to generate sub-indices. It is important to note that it is impractical, if not impossible, to assess all the ecosystem services in a study area. This analysis only attempts to include as many major ecosystem services as possible using the best available data in our study area.

As the term implies direct ecosystem benefits are those that are used directly in generating human well-being. For example, agricultural products are provisioning services that provide direct benefits from agricultural ecosystems. Other services contribute indirectly to human well-being. Sometimes indirect benefits are only one step removed from direct benefits (i.e., first-order indirect benefits) and sometimes they are more distantly linked (i.e., secondary or more distant indirect benefits). For example, local households do not directly partake in the ecotourism activities but they one-step indirectly benefit from the cultural services of ecotourism through providing transportation, food and accommodation services to eco-tourists. But ecotourism may also enhance the development of infrastructure (e.g., road construction), create more job opportunities, and thus provide indirect benefits several steps removed from the cultural services. Generally, the challenge in identifying benefits for CBA is to separate the genuine indirect effects from those that are double accounting [54]. Usually, if there is not a strong rationale, only direct benefits and costs are included to avoid double accounting [33]. However, in our study area, first-order indirect benefits capture an important part of benefits from ecosystem services and the inclusion of them do not cause double-accounting (Table S1). As a first approximation, here we included direct benefits and first-order indirect benefits in our calculations because these captured the majority benefits in our study area (Table S1). We adapted the MA classification for types of related ecosystem services (Table S1) to make it appropriate for our study area. For some specific services, the classification may differ from what would be appropriate in other areas. But this does not affect the comparisons using the overall index and sub-indices of IDES, which are based on the generalizable MA framework.

Some households obtained negative net benefits in agricultural operating income when the total gross agricultural income was lower than total agricultural expenditure, due to pests, diseases, natural disasters (e.g., storms and landslides), and/or low prices of agricultural products. Most income from ecosystem services comes from provisioning services, but some households also have income from ecotourism, which we categorized as benefits related to cultural services, and income from PES programs, which we categorized as benefits related to regulating services (Table S1).

The benefits that households obtained from ecosystems include not only the benefits reflected in their income, but also the avoided costs not reflected in their expenditures. Two major items of avoided costs were assessed here. One is the reduced electricity fees through a subsidized electricity price. Because the conservation of forests also dramatically conserves watersheds in our study area, local households were given a reduction of electricity price of 0.07 yuan per kilowatt-hour in both 1998 and 2007 (yuan: Chinese Currency, 1 USD = 7.52 yuan as of 2007). Thus, the avoided electricity fees could be calculated by multiplying their consumed electricity amount and the reduced price. Another item of avoided cost is from fuelwood collection for energy use. Households would need to pay for alternative energy sources (e.g., electricity, coal) if they do not collect fuelwood. Because households need to spend labor collecting fuelwood, in the past when one household did not have enough laborers in the fuelwood collection season, one might exchange laborers or hire laborers from other households. Thus, the monetary value of collected fuelwood can be estimated as the market value of the labor spent on collecting it. In our household survey, we measured the collected amount of fuelwood and total labor spent in collecting it. We then calculated the shadow price of fuelwood (approximately 0.10 yuan per kilogram in 1998 and 0.20 yuan per kilogram in 2007). Data for each household on each of these sources of net income and avoided costs were then used to construct the index system. The dataset used for this study is provided in the Supporting Information (Data file S1).

### 3.3 Results of the index system

Table 1 showed the results of net benefits from different sources and the overall IDES and corresponding sub-indices in both 1998 and 2007. Our results showed a dramatic increase of net benefits from all categories of ecosystem services and socioeconomic activities. From 1998 to 2007, the total net benefit from ecosystem services has increased from an average of approximately 1,723 yuan to 12,972 yuan (both values were in present values for 1998). Meanwhile, from 1998 to 2007, the total net benefit from socioeconomic activities also has dramatically increased from an average of approximately 2,456 yuan to 12,350 yuan.

**Table 1:** Net benefits, overall IDES and sub-indices in 1998 and 2007.

Net benefits/Indices	1998		2007	
	Mean (S.D.)	Range (Minimum: Maximum)*	Mean (S.D.)	Range (Minimum : Maximum)
Net socioeconomic benefit (yuan)	2456.38 (3315.50)	(0∶16,600)	12,350.10 (21,027.75)	(0∶186,046)
Net benefit from provisioning services (yuan)	2308.77 (2506.89)	(−2671∶13,676)	8544.97 (14,063.43)	(−2620∶107,003)
Net benefit from regulating services (yuan)	77.60 (92.88)	(0∶544)	2900.64 (2003.59)	(0∶10,448)
Net benefit from cultural services (yuan)	3.33 (44.72)	(0∶600)	1526.10 (13,476.34)	(0∶177,626)
Total net benefit from ecosystem services (yuan)	2389.71 (2527.22)	(−2620∶14,182)	12971.70 (19,043.19)	(−27∶181,801)
Sub-index for provisioning services	0.4131 (0.8627)	(−7.0351∶0.9973)	0.3754 (0.3131)	(−0.1750∶1)
Sub-index for regulating services	0.0340 (0.0714)	(0∶0.7518)	0.2112 (0.2026)	(0∶1)
Sub-index for cultural services	0.0003 (0.0038)	(0∶0.0513)	0.0257 (0.1143)	(0∶0.8568)
Overall IDES	0.4473 (0.8237)	(−6.9019∶1)	0.6123 (0.3055)	(−0.0015∶1)

Notes:

Monetary values for net benefits in 2007 were discounted into present values of 1998 for comparison.

* Negative value of an index means that the gross benefit from ecosystem services is lower than the sum of costs from ecosystem dis-services and costs of generating the corresponding ecosystem services.

Table 1 also showed that the overall index of households' dependences on ecosystem services has increased from approximately 0.42 in 1998 to 0.61 in 2007. The average overall IDESs were 0.45 in 1998 and 0.61 in 2007, indicating that approximately 45% and 61% of total net benefits to households came from ecosystem services in 1998 and 2007, respectively. Approximately 54% and 63% households had an overall IDES larger than 0.50, and 9% and 16% households had an overall IDES of 1.00 in 1998 and 2007, respectively. Overall these results suggested that most households in our study area were highly dependent on ecosystem services and some were essentially completely dependent on them.

The percent of households obtained positive net benefits from provisioning services were 89% and 85% in 1998 and 2007, respectively. Almost all households benefited from regulating services in both 1998 and 2007. Perhaps most interesting, almost all households in 2007 acquired positive net benefits from regulating services through the PES programs (i.e., NFCP, GTGP, and GTBP). These programs were the major reason for the dramatic increase of net benefits from regulating services. However, almost no household in 1998 and only 11% households obtained positive net benefits from cultural services such as ecotourism.

### 3.4 Advantages and applications of the index system

Our IDES is better than the agricultural income share in reflecting households' dependences on ecosystem services. Agricultural income share, or the ratio of agricultural income to total income, is a commonly used indicator that can approximately reflect a rural household's dependence on ecosystem services. Although the proposition that the poor are more dependent on ecosystem services is rarely examined quantitatively, it is a widely accepted notion [3], [12]. Here, we compared the overall IDES with agricultural income share by examining their relation to overall household income. Our results suggested that the overall IDES were negatively associated with household income in both 1998 and 2007 (Table 2).That is, higher income households make less use of ecosystem services. These results confirmed the common view that low incomes households are more dependent on ecosystem services. However, the association of household income with agricultural income share was significant only in 1998 but not in 2007 (Table 2). These results indicated that our overall IDES was better than the agricultural income share as a measure of rural households' dependences on ecosystem services. In our study area, income had become decoupled from income share from agriculture but not from use of ecosystem services by our second survey.

**Table 2:** Comparison of overall IDES and agricultural income share for their associations with gross household income.

	Household income in 1998	Household income in 2007
Agricultural income share	−0.355***	−0.012
Overall IDES	−0.194**	−0.405***

Notes:

Numbers are Spearman's rhos. Total samples are the same 180 randomly sampled households across years.

* p<0.05;

** p<0.01;

*** p<0.001.

Comparing the results of the index system in 1998 and 2007 (Table 1), the reasons that IDES is a better measure than agricultural income share are easy to see. In 1998, most of the household income was from agriculture, which was classified as benefits related to provisioning services (Table S1). The overall IDES was almost equivalent to the sub-index for provisioning services (Table 1) and thus was similar to agricultural income share in reflecting a household's dependence on ecosystem services in 1998. In 2007, household income sources became more diverse and included many non-agricultural items (Table S1). Therefore, unlike the overall IDES, agricultural income share no longer accurately reflected households' dependences on ecosystem services.

These results demonstrate some of the uses of the index system. We further illustrated its applications by examining how the benefits of ecosystem services were unequally available to households. Because ecosystem services flow from natural capital, households who possess substantial access to natural capital should obtain more benefits from ecosystems and thus would be more dependent on ecosystem services than those with less access to natural capital. The positive associations between indices of dependence on ecosystem services and the area of cropland supported this argument (Table 3). Although poverty is often defined in terms of low access to financial capital, households in financial poverty often have limited access to human, manufactured, and social capital. Thus, the proposition that the poor depend more on ecosystem services may be generalized from poverty in financial capital to poverty in human, manufactured, and social capital. Table 3 supported such negative associations between indices of high dependence on ecosystem services and a number of measures of low access to different forms of capital.

**Table 3:** Regression of sources of variation on overall IDES.

	Variable	IDES 1998	IDES 2007
Natural capital	Area of cropland (Mu, 1 Mu = 1/15 ha)	0.020 (0.014)	0.042*** (0.007)
Human capital	Household size	−0.077 (0.049)	−0.037* (0.016)
	Number of laborers	−0.070 (0.056)	−0.080*** (0.018)
	Average education of adults (year)	−0.032† (0.017)	−0.032*** (0.009)
	Average age of adults (year)	0.011* (0.005)	0.008*** (0.002)
Financial capital	Household income (yuan, log)	0.071 (0.075)	−0.152*** (0.025)
	Per capital income (yuan, log)	0.143 (0.095)	−0.126*** (0.028)
Manufactured capital	Type of house (0 for low quality non-concrete sheds and 1 for high quality concrete house)	−0.022 (0.139)	−0.189*** (0.048)
	Distance to the main road (meter, log)	−0.029 (0.027)	0.042*** (0.010)
Social capital	Social ties to local township and reserve level officials (0: low; 1: high).	0.065 (0.129)	−0.188** (0.065)
Spatial heterogeneity	Township (0: Gengda; 1: Wolong)	0.098 (0.133)	−0.101* (0.047)

Numbers outside and inside parentheses are coefficients and robust standard errors of bivariate regressions, respectively. Dependent variables are overall IDES in 1998 and 2007, respectively.

Notes:

Total samples are the same 180 randomly sampled households across years.

† p<0.01;

* p<0.05;

** p<0.01;

*** p<0.001.

It should be noted that the relationship between dependence on ecosystem services and lack of access to forms of capital can change rather rapidly. In 1998, the local economy at Wolong Nature Reserve was relatively closed and mainly relied on agriculture. Since 2000, the NFCP, GTGP, GTBP, and tourism development have led to more non-farm income. The local economy became more open. For example, thousands of tourists visit Wolong Nature Reserve every year to view giant pandas. As a result, local farmers began to grow vegetables selling to outside markets, and some young farmers enrolled their cropland in GTGP and GTBP and migrated to urban areas for work. These were the reasons why some indicators of capital such as the distance to the main road, area of cropland, social ties were not as important in 1998 as they were in 2007 (Table 3). Furthermore, most households in 1998 lived in low quality wooden or stone sheds, while in 2007, with more money available, some of them lived in high quality concrete houses. Households actively strategize how to substitute one form of capital for another in order to achieve access to needed resources and enhance well-being [55].

## Discussion and Conclusions

This proposed index system is a step forward in quantifying human dependence on ecosystem services. As mentioned above, it is impractical, if not impossible, to capture all ecosystem services in any study. However, we have implemented the index system with existing household survey data and were able to capture major ecosystem services in our initial estimates. Our results suggest that such an approximation is a viable approach that reveals useful information on human dependence on ecosystem services. The overall index and its sub-indices can reveal the general pattern of households' dependences on ecosystem services, and the variations across time, space, and different levels of access to multiple forms of capital.

Given the fact that different individuals, households and communities rely on different ecosystem services, in practice it is likely that the measurement of benefits, costs and IDES will depend on relatively detailed data that is collected with an understanding of the local context. To facilitate comparisons across time, space and different institutional levels, it is necessary that different studies use a common platform such as the generally accepted MA framework for classification. While it will usually not be possible to capture all benefits and costs associated with ecosystems services, care should be taken to accurately estimate the most important benefits and costs in the local context in order to construct overall IDES estimates. To avoid misinterpretation in some circumstances (e.g., the effect of Integrated Conservation and Development Projects on changes in households' dependence on ecosystem services), such comparisons should not only focus on the overall IDES index but also consider the sub-indices as well as the structure of dependence on ecosystem services (i.e., the distribution of three sub-indices).

It should be noted that IDES measures the relative importance of ecosystem services, with comparison to other socioeconomic activities, in providing benefits directly and indirectly to humans. If one wants to compare the absolute values of benefits from ecosystem services across different areas, one should use the net benefits obtained from ecosystem services, which are also provided through construction of the IDES system.

It should also be noted that there is a substantial difference between being dependent on ecosystem services and being dependent on PES. The PES program compensates some of the forgone benefits that local households enjoyed before the implementation of conservation policies. But the PES program does not necessarily compensate all the forgone benefits. For example, in our study area, the main purpose of payments from NFCP is to protect natural forests. As a result payments from NFCP mostly compensate the forgone provisioning (e.g., timber harvest) services whereby regulating services (e.g., soil erosion control, carbon sequestration, and water conservation) are increased. Cultural services (e.g., recreation and ecotourism) are not included. In our study, we therefore included benefits from ecotourism which are not captured in the PES from the NFCP.

Using the overall IDES, we confirmed the proposition that the poor are more dependent on ecosystem services, and thus are more vulnerable to degradation or decline of the corresponding ecosystem services. More importantly, we generalized this proposition to those disadvantaged groups who possess less access to multiple forms of capital (i.e., human, financial, manufactured, and social capital) and found they too were more dependent on ecosystem services than the affluent.

Although we demonstrated the construction of the IDES system and its applications based only on data from the Wolong Nature Reserve, the conceptual basis of IDES and methodology we used were designed to be generalizable. While we examined households, the unit of analysis could range from individuals to communities, regions, and nations. Our analysis here is a proof of the concept of IDES. Further elaborations are warranted and could potentially improve the estimates of human dependence on ecosystem services.

We believe the IDES index system presented here has some major advantages to advance the understandings of linkages between ecosystem services and human well-being and support decision-making. First, this paper empirically demonstrated how the index system could better reflect human dependence on ecosystem services than the other commonly used indicator (i.e., agricultural income share) at the household level. Second, the index system provides both a composite index and sub-indices. This allows the quantitative analysis of the structure of human dependence on ecosystem services and the quantitative examination of the interwoven linkages between different types of ecosystem services and different components of human well-being. Future studies could combine IDES with indicators of indirect drivers, direct drivers, and human well-being to construct integrated models based on the MA framework to better understand complex interactions among human and natural components (e.g., to assess how human dependence on ecosystem services may affect human well-being). The improved understanding may help to develop theories on the complexity of CHANS and inform decision making in a rapidly changing global environment. Third, the improved understanding of linkages between ecosystem services and human well-being, if integrated into decision-making, may avoid some inappropriate strategies that aggravate the marginalization of disadvantaged groups. This in turn could reduce the pressure these groups place on ecosystems to obtain services critical to them. However, a distinction should be made: dependence on ecosystem services is not equivalent to pressure on ecosystems because there are often sustainable ways to extract ecosystem services. Acquisitions of regulating and cultural services (e.g., air purification and ecotourism) are often non-consumptive, while many uses of provisioning services (e.g., timber, fuelwood) are often consumptive and may or may not be sustainable. The reduction of pressure or impacts on ecosystem services can be realized through reduction of overall dependence on ecosystem services or through a shift of the structure of dependence to different types of ecosystem services such as a shift from high dependence on provisioning services to high dependence on regulating and cultural services. It can also be achieved by extracting provisioning services in ways that do not harm the ecosystem. Fourthly, improved understanding may also enhance the effectiveness and long-term viability of conservation and development programs by targeting priority population groups such as those with limited access to capital and high dependence on provisioning services. Finally, such understanding could draw stakeholders' attention to the unmanaged risks and unrealized opportunities associated with ecosystem service changes. Climate change and other global changes are causing rapid shifts in ecosystem structure and function and may threaten continued flow of services to those most dependent upon them. Taking our study area as an example, conservation and development efforts such as NFCP, GTGP, GTBP, and tourism development have already reduced many households' dependences on provisioning services; however, the very uneven distribution of benefits from ecosystem services may create potential risks and impede the future success of such policies.

## Supporting Information

Table S1 **Detailed classification of household net income and avoided costs by type of related ecosystem services.**
(DOC)

Data file S1 **Dataset used in this study.**
(ZIP)
