# Supplementary material for: Going Beyond the Millennium Ecosystem Assessment: An Index System of Human Dependence on Ecosystem Services
Source: PLoS One. 2013 May 22;8(5):e64581. doi: 10.1371/journal.pone.0064581 (PMC3661539; doi:10.1371/journal.pone.0064581)
Supplement: Table S1 — Detailed classification of household net income and avoided costs by type of related ecosystem services. (DOC) [file pone.0064581.s001.doc]

Table S1. Detailed classification of household net income and avoided costs by type of related ecosystem services.

| Category | Sub-category | Item | Type of related ecosystem services^*^ |
| --- | --- | --- | --- |
| Operating Income | Crop income | INC101: Cabbage | P0 |
|  |  | INC102: Radish | P0 |
|  |  | INC103: Potato | P0 |
|  |  | INC104: Corn | P0 |
|  |  | INC105: Other crops | P0 |
|  | Animal husbandry income | INC106: Bacon | P1 |
|  |  | INC107: Pig | P0 |
|  |  | INC108: Goat | P0 |
|  |  | INC109: Cattle | P0 |
|  |  | INC110: Yak | P0 |
|  |  | INC111: Horse | P0 |
|  |  | INC112: Poultry and eggs | P0 |
|  |  | INC113: Honey bee | P0 |
|  |  | INC114: Other husbandry | P0 |
|  | NTFPs income | INC115: Non-timber Forest Products (NTFPs) | P0 |
|  | Other agricultural operating income | INC116: Other agricultural operating income | P0 |
|  | Non-agricultural operating income | INC117: Restaurants and hotels | C1 or NA^†^ |
|  |  | INC118: Ecotourism | C1 or NA^†^ |
|  |  | INC119: Transportation | C1 or NA^†^ |
|  |  | INC120: Contract work | NA |
|  |  | INC121: Other small businesses | C1 or NA^†^ |
| Wage Income |  | INC201: Wage and bonus | NA |
|  |  | INC202: Local labor income | NA |
|  |  | INC203: Migrant labor income | NA |
| Property Income | Land and housing rents | INC301: Land and housing rents | C1 or NA^†^ |
|  | Other property income | INC302: Interest income | NA |
|  |  | INC303: Land acquisition compensation | NA |
|  |  | INC304: Other rents | NA |
| Transfer Income | Gift income from relatives and friends | INC401: Gift income from relatives and friends | NA |
|  | Payments for ecosystem services (PES) income | INC402: Natural forest conservation program (NFCP) | R0 |
|  |  | INC403: Grain-to-Green program (GTGP) | R0 |
|  |  | INC404: Grain-to-Bamboo program (GTBP) | R0 |
|  | Social security Benefits | INC405: Low income subsidy | NA |
|  |  | INC406: Pension | NA |
|  |  | INC407: Other subsidies | NA |
| Other Income |  | INC501: Remaining other socioeconomic income | NA |
| Avoided costs |  | Fuelwood for energy use | P0 |
|  |  | Subsidized electricity fees due to watershed conservation | R1 |

Notes:

^*^: Letters P, R, C, and NA represent provisioning services, regulating services, cultural services, and benefits unrelated to ecosystem services respectively. The digits “0” and “1” after “P, R or C” represent direct and first-order indirect ecosystem services, respectively. In our case, PES programs were designed mainly for regulating services (e.g., water conservation, soil erosion control, carbon sequestration, and air purification). Thus, we classified PES as benefits related to regulating services. Dis-services and costs of delivering ecosystem services have already been included in the net income data, which were best available approximations of net benefits here. The data and detailed description of each variable are provided in the Supporting Information file.

^†^: For each household, if the benefit is related to ecotourism, it is included as a benefit related to cultural services; or else, it is regarded as a benefit unrelated to ecosystem services.
